# Clinical Impact of Combined Modified Glasgow Prognostic Score and C-Reactive Protein/Albumin Ratio in Patients with Colorectal Cancer

**DOI:** 10.3390/diagnostics10110859

**Published:** 2020-10-22

**Authors:** Woosung Son, Su-Jin Shin, Su Hyeong Park, Soo Kyung Lee, Eun Jung Park, Seung Hyuk Baik, Kang Young Lee, Jeonghyun Kang

**Affiliations:** 1 Department of Surgery, Gangnam Severance Hospital, Yonsei University College of Medicine, Seoul 06273, Korea; ANDYSON@yuhs.ac (W.S.); KZPGG@yuhs.ac (S.H.P.); LSK11@yuhs.ac (S.K.L.); camp79@yuhs.ac (E.J.P.); whitenoja@yuhs.ac (S.H.B.); 2 Department of Pathology, Gangnam Severance Hospital, Yonsei University College of Medicine, Seoul 06273, Korea; CHARM@yuhs.ac; 3 Department of Surgery, Severance Hospital, Yonsei University College of Medicine, Seoul 03722, Korea; kylee117@yuhs.ac

**Keywords:** modified glasgow prognostic score, C-reactive protein/albumin ratio, colorectal cancer, survival, integrated area under the curve

## Abstract

The prognostic impact of the combination of the modified Glasgow prognostic score (mGPS) and C-reactive protein/albumin ratio (CAR) in colorectal cancer (CRC) is unclear. We aimed to investigate the clinical usefulness of this combination as a predictor of survival in CRC patients. We retrospectively evaluated 769 CRC patients who had undergone surgery between January 2006 and March 2014. The CAR and mGPS within 1 month postoperation were examined. The integrated area under the curve (iAUC) was compared among mGPS, CAR, and the combined classification (CC). The optimal CAR cut-off for discriminating overall survival was 0.14. Based on this cut-off, the mGPS 0 group was divided into the mGPS 0 with low CAR and the mGPS 0 with high CAR groups, whereas all mGPS 1 and 2 patients were classified into the high CAR group. CC was an independent prognostic factor, and its iAUC value (0.587, 95% CI 0.553–0.624) was superior to those of the mGPS (0.544, 95% CI 0.516–0.576) (bootstrap iAUC mean difference = 0.043; 95% CI = 0.015–0.072) and CAR (0.578, 95% CI 0.545–0.613) (bootstrap iAUC mean difference = 0.009; 95% CI = 0.002–0.017), respectively. In conclusion, the combination of mGPS and CAR has a synergistic effect and has a higher prognostic accuracy than mGPS or CAR alone in patients with CRC.

## 1. Introduction

Colorectal cancer (CRC) is the third most commonly diagnosed cancer worldwide, accounting for 11% of all cancer diagnoses [1]. Approximately 1.8 million new cases of CRC were diagnosed in 2018, and CRC is the most commonly diagnosed cancer among men in 10 of the 191 countries worldwide [1]. CRC also ranks third in terms of the estimated crude and age-standardized cancer mortality rates in South Korea [2].

The American Joint Committee on Cancer (AJCC) staging system has been used as the standard for decision-making for treatment, and the AJCC stage has been known as an independent factor for distinguishing patients based on predicted survival outcomes [3]. However, there is a limitation that the AJCC stage does not sufficiently reflect the patient’s prognosis [4,5,6]. Thus, many investigators continue to attempt to predict prognosis using molecular or genomic data [7,8,9]. However, such methods are not cost-effective, and thus, routinely available prognosticators that can help clinical decision-making need to be identified.

Systemic inflammation has been known as an important predictor of survival in patients with CRC [10]. The modified Glasgow prognostic score (mGPS) is one of the most widely validated prognostic tools, with numerous studies reporting that a high mGPS is associated with adverse patient outcomes in various cancers, including CRC [11,12]. The C-reactive protein (CRP)/albumin ratio (CAR) was recently introduced as an alternative prognostic marker in patients with CRC [13]. In this context, a recent study simultaneously investigated the prognostic values of mGPS and CAR in the same cohort and identified both as independent prognostic factors [14].

However, despite being derived from the same criteria involving circulating CRP and albumin levels, the mGPS and CAR may have different clinical implications. Ishizuka et al. reported that CAR can be used to further classify CRC patients with an mGPS of 0 and 1 into two separate groups, with these groups showing different survival outcomes. This suggests the possibility of CAR and mGPS being complementary [13]. However, the clinical significance of the combination of these two indicators is yet to be clarified, and it remains unclear whether their combination has higher accuracy for stratifying the prognosis of patients with CRC.

Thus, this study aimed to investigate the clinical usefulness of the combination of CAR and mGPS in comparison to that of mGPS or CAR alone for predicting the survival of patients with CRC.

## 2. Materials and Methods

### 2.1. Study Design and Patient

**Figure 1:**
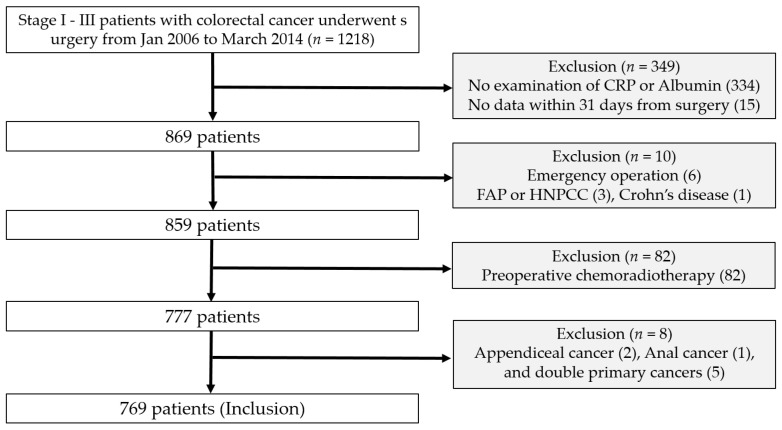
Flow diagram of patients. CRP: C-reactive protein, FAP: Familial adenomatous polyposis, and HNPCC: Hereditary nonpolyposis colorectal cancer.

This retrospective, single-center study was approved by the Institutional Review Board of our hospital (approval number: 3-2020-0144; 27 May 2020). The need for informed consent was waived owing to the retrospective nature of the study. The subjects were patients with stage I–III CRC who underwent curative resection at Gangnam Severance Hospital, Yonsei University College of Medicine between January 2006 and March 2014. The inclusion criteria were as follows: (1) pathologically confirmed CRC, (2) surgery with curative intent, and (3) measurement of CRP and albumin levels within 31 days of surgery. The exclusion criteria were as follows: (1) emergency operations, (2) history of familial adenomatous polyposis or hereditary nonpolyposis colorectal cancer, (3) history of inflammatory bowel diseases such as ulcerative colitis and Crohn’s disease, (4) preoperative radiotherapy or chemoradiotherapy, and (5) double primary cancers (Figure 1).

### 2.2. Follow-Up Protocol

Follow-up involved outpatient clinic visits every 3 months for 3 years and every 6 months thereafter until 5 years. The serum carcinoembryonic antigen (CEA) level was routinely measured at each follow-up visit. Abdominopelvic computed tomography (CT) was performed at an average interval of 6 months. Colonoscopy, chest CT, or ^18^F-fluorodeoxyglucose positron-emission tomography was performed as indicated according to the surgeon’s discretion. Patient follow-up lasted until the cut-off date (October 2019) or death. The patients were followed up for a median of 91 months (interquartile range, 67–115 months).

### 2.3. mGPS, CAR, and the Combination of These Two Parameters

The mGPS was determined as follows: mGPS 0 = CRP ≤ 10 mg/L, mGPS 1 = CRP >10 mg/L and albumin ≥3.5 g/dL, and mGPS 2 = CRP >10 mg/L and albumin <3.5 g/dL. Meanwhile, the CAR was calculated as follows: CAR = serum CRP level (mg/dL)/serum albumin level (g/dL) [15]. The optimal cut-off value for the CAR was defined as the reference value that could produce the largest χ2 in the Mantel–Cox test [16]. Each mGPS was allocated to one of the two groups based on this cut-off value of the CAR. This new combination was denoted as the combined classification (CC) throughout the rest of this study.

### 2.4. Statistical Analysis

All statistical analyses were performed using R v. 3.6.3 (R-project, Institute for Statistics and Mathematics, Vienna, Austria). Differences in the distribution of categorical variables were compared using the chi-square test, while those in the distribution of continuous variables were analyzed using the Kruskal–Wallis test. Overall survival (OS) was estimated using the Kaplan–Meier method and compared using the log-rank test. The Cox proportional hazard model was used to estimate HRs and 95% CIs for factors associated with OS. Significant factors (*p* < 0.1) in the univariate Cox regression analysis were entered into multivariate analysis models.

The prognostic capabilities of the mGPS, CAR, and CC were compared by generating time-dependent ROC curves and by calculating the estimated AUC. Time-dependent ROC curve analysis is an extension of ROC curve analysis and assesses the discriminatory power of continuous markers for time-dependent disease outcomes. In addition to visually comparing the ROC curves, the AUC can be calculated. The integrated AUC (iAUC) is a weighted average of the AUC across a specific period. This is a way of measuring predictive ability of the model during a period of follow-up. A higher iAUC indicates a better prognostic performance. The bootstrapping method was used to calculate the differences and 95% confidence interval [17]. Harrell’s concordance index (C-index) indicated the model’s ability to accurately predict specific outcomes and discrimination was evaluated using C-index [18]. The value of C-index ranged from 1 to 0.5, which represents a perfect prediction and a random chance to correctly predict the outcomes, respectively. The performance was compared by measuring the discrimination ability using bootstraps with 1000 resamples. A two-sided *p* < 0.05 was considered statistically significant.

## 3. Results

### 3.1. Patient Characteristics

#### Comparison of Clinicopathologic Factors According to mGPS

A total of 769 patients who underwent potentially curative resection for CRC were included in the analysis (Table 1). The most common mGPS score was 0 (*n* = 609, 79.2%), followed by 1 (*n* = 122, 15.8%), and 2 (*n* = 38, 4.9%). Age, American Society of Anesthesiologist (ASA) grade, CEA levels, tumor location, tumor size, histologic grade, complications, number of retrieved lymph nodes (LNs), and AJCC stage were independently associated with the mGPS. Meanwhile, sex, body mass index (BMI), lymphovascular invasion (LVI), and chemotherapy were not independently associated with the mGPS. A higher mGPS was more likely to be associated with a higher CAR than a low mGPS.

### 3.2. Cut-off Value of CAR

**Figure 2:**
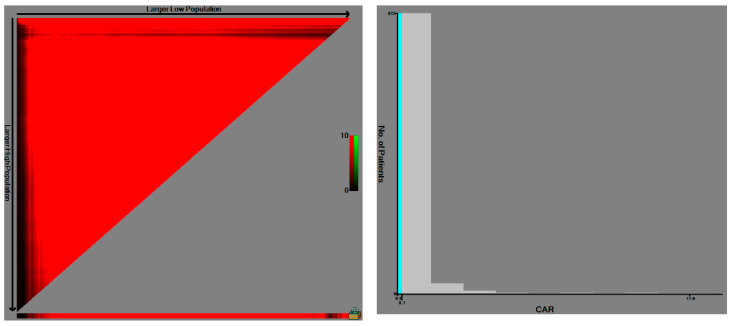
Determining the cut-off value of the CAR using the X-tile program. The variable color points in the X-tile plots represent associated strength at each split from low (dark, black) to high (bright, red, or green). Red indicates an inverse association between the expression levels and survival of the variables, while green indicates a direct association. We defined optimal cut-off value as the value that produced the largest χ^2^ in the Mantel–Cox test, which was set as 0.14.

The cut-off value of the CAR that produced the largest χ2 in the Mantel–Cox test was 0.14 (Figure 2).

### 3.3. Combined Classification and Survival

**Figure 3:**
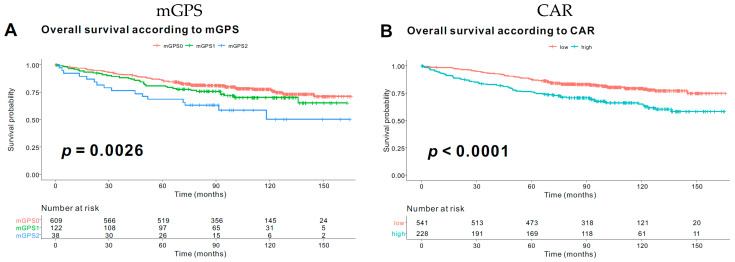

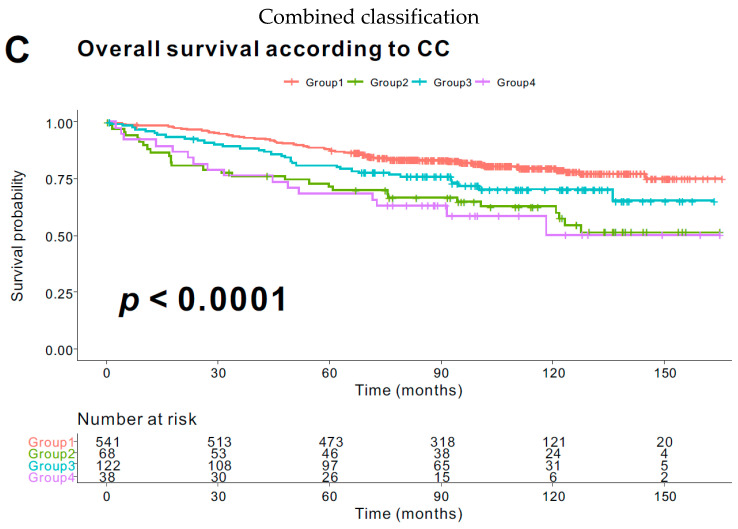
Kaplan–Meier curves of overall survival according to the mGPS, CAR, and combination classification.

Based on this value, the patients were divided into two subgroups in the succeeding survival analysis as follows: CAR-low (*n* = 541, 70.3%) and CAR-high (*n* = 228, 29.6%). In the Kaplan–Meier survival analysis, both the mGPS and CAR were significantly associated with OS. For the combined classification (CC) (i.e., combination of mGPS and CAR), all patients were initially re-classified into six groups (mGPS: 0, 1, 2; and CAR: low and high). However, no patient satisfied the “mGPS 1 with low CAR” and “mGPS 2 with low CAR” classifications. Therefore, only four CC groups were included in the survival analysis, and the Kaplan–Meier survival curves showed significant differences among the four groups (*p* < 0.0001) (Figure 3).

### 3.4. Univariate Analysis

In the univariate analysis, age (*p* < 0.001), BMI (*p* = 0.018), CEA level (*p* < 0.001), tumor size (*p* = 0.045), LVI (absent vs. present, *p* < 0.001), number of retrieved LNs (*p* = 0.017), and AJCC stage (I vs. II, *p* = 0.006; I vs. III, *p* < 0.001) were significantly associated with OS (Table 2). The mGPS 2 group showed significantly worse OS than the mGPS 0 group (hazard ratio (HR) = 2.32, 95% confidence interval (CI) = 1.38–3.90, *p* = 0.001), whereas there was no significant difference in OS between the mGPS 1 and mGPS 0 groups (HR = 1.35, 95% CI = 0.92–1.96, *p* = 0.118). The CAR-high group also tended to show a poorer survival rate than the CAR-low group (HR = 1.97, 95% CI = 1.47–2.64, *p* < 0.001) (Table 2). Among the four CC groups, Group 1 (mGPS 0 and low CAR) showed a significantly better OS than the other three groups (vs. Group 2 (mGPS 0 and high CAR): HR = 2.43, 95% CI = 1.60–3.69, *p* < 0.001; vs. Group 3 (mGPS 1 and high CAR): HR = 1.54, 95% CI = 1.04–2.27, *p* = 0.028; vs. Group 4 (mGPS 2 and high CAR): HR = 2.65; 95% CI = 1.56–4.49, *p* < 0.001).

### 3.5. Multivariate Analysis

First, we evaluated prognostic significance among mGPS, CAR, and CC. Using multivariate analysis and including only three parameters, CC remained the only significant factor (data not shown). Thus, CC was included in the final multivariate analysis adjusting with clinicopathological variables. In the multivariate survival analysis, CC was associated with OS, independent of sex, age, BMI, CEA, complications, number of retrieved LNs, and AJCC stage (Group 1 vs. Group 4, HR = 1.90, 95% CI = 1.09–3.30, *p* = 0.022) (Table 3).

### 3.6. Time Dependent ROC among mGPS, CAR, and CC 

**Figure 4:**
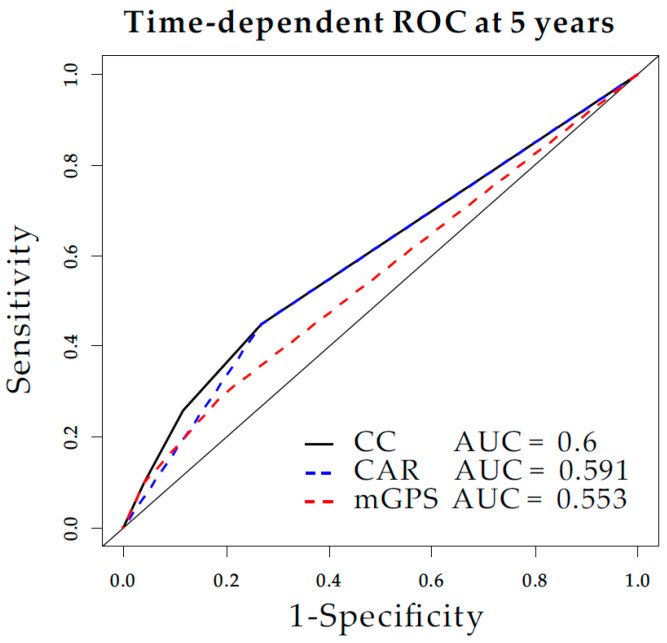
Time-dependent receiver operating characteristic (ROC) among the mGPS, CAR, and CC.

Time-dependent receiver operating characteristic (ROC) curve of mGPS, CAR, and CC at 5 year revealed Area Under the Curve (AUC) value as 0.553, 0.591, and 0.6 respectively (Figure 4).

### 3.7. Integrated AUC and C-Index Comparison among mGPS, CAR, and CC

**Figure 5:**
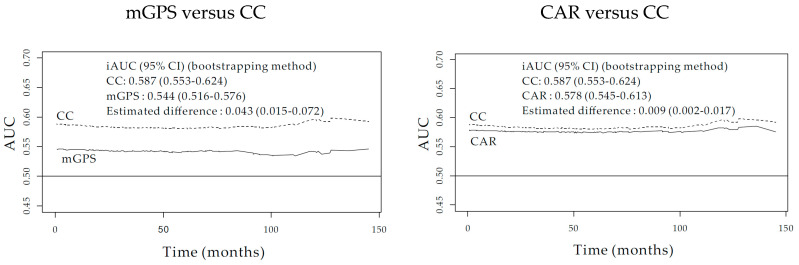
Comparison of integrated AUC among the mGPS, CAR, and CC.

The integrated AUC value (0.587, 95% CI = 0.553–0.624) was superior to those of the mGPS (0.544, 95% CI = 0.516–0.576) (bootstrap iAUC mean difference = 0.043, 95% CI = 0.015–0.072) and CAR (0.578, 95% CI = 0.545–0.613) (bootstrap iAUC mean difference = 0.009, 95% CI = 0.002–0.017), respectively, throughout the observation period (Figure 5). The C-index of CC (0.587, 95% CI = 0.549–0.628) showed superior discriminatory power to that of the mGPS (0.546, 95% CI = 0.515–0.580) (bootstrap mean difference = 0.040; 95% CI = 0.013–0.071) and CAR (0.578, 95% CI = 0.542–0.617) (bootstrap mean difference = 0.008; 95% CI = 0.001–0.016), respectively.

### 3.8. Clinical Impact of Adding CC into AJCC Stage

Harrell’s concordance index was compared between stage and stage plus CC. C-index of stage plus CC showed better discriminatory power to that of stage only (stage + CC; 0.651, 95% CI = 0.612–0.689 vs. Stage; 0.615, 95% CI = 0.579–0.65, *p* = 0.0056) (Table 4).

## 4. Discussion

Both the mGPS and CAR are individually established prognostic markers in CRC, but the prognostic impact of their combination is yet to be clarified. This study demonstrated that the combination of these two parameters has higher predictive accuracy for survival than the mGPS or CAR alone in patients with CRC.

Systemic inflammatory response is an established prognostic factor regardless of disease stage [19]. Although the detailed pathogenesis is still unclear, a marked systemic-inflammatory response is associated with decline in the patients’ nutritional, functional, and immunological status [12]. As an indicator of systemic inflammation that predicts the survival of cancer patients, the mGPS is an effective prognostic indicator in various diseases including cancer [20]. CRP, an acute-phase protein that is synthesized in the liver along with other inflammatory cytokines [21], has been reported to possibly lead to tumor progression by inducing the production of inflammatory cytokines and chemokines [22]. Albumin accounts for half of the plasma protein content, and albumin levels can indicate the nutritional status and inflammatory response. As an acute-phase protein, its levels or ratios are also involved with other inflammatory indices. Naturally, these two markers may represent a balance between the inflammation and nutritional status. Accordingly, an increased CAR or mGPS has been known to indicate poor prognosis.

A recent study that simultaneously analyzed the CAR and mGPS in the same cohort showed that they are independent prognostic factors of cancer-specific survival and OS in patients with colon cancer [14]. However, there are several serum markers of systemic inflammatory status, and the mechanism by which they impact prognosis may differ. Thus, each marker is applied differently in the clinical setting. Accordingly, it may be meaningful to combine established nutrition- and inflammation-related prognostic factors to expand their independent prognostic capabilities and improve the accuracy of prognostic prediction. Laird et al. demonstrated that the combination of the mGPS and performance status had higher prognostic accuracy in patients diagnosed with advanced cancers [23]. Inamoto et al. also reported that the combination of the GPS and neutrophil-to-lymphocyte ratio (NLR) can be used to effectively stratify patient outcomes, with patients with an NLR of <2.05 and a GPS of 0 having a remarkably better prognosis [24].

The mGPS and CAR are easy to use in combination because they are both assessed using the same laboratory variables. A previous study showed that patients with an mGPS of 0 and 1 could be further classified into two different groups based on the cut-off value of the CAR [13], suggesting that the mGPS and CAR act as complementary variables. However, the synergistic effect of combining these two parameters has not been fully investigated. Our study demonstrated that combining these two parameters could provide better predictive power throughout the time points, and CC can, thus, be used as an effective inflammation-based prognostic marker instead of mGPS or CAR alone. The mGPS 0 and low CAR group showed a better prognosis than the other groups. Such characteristics may be an indication for selecting the 3-month FOLFOX chemotherapy regimen over the conventional 6-month treatment regimen in patients with stage III colon cancer or in patients with stage II colon cancer who did not receive adjuvant chemotherapy. However, further prospective studies should be performed to prove these hypotheses.

Although several studies have provided evidence supporting the prognostic value of the mGPS, some studies have reported conflicting findings. Son et al. reported that the mGPS does not predict OS (mGPS 2 vs. 0–1, HR = 2.217, 95% CI = 0.716–6.864, *p* = 0.167) [25]. Chan et al. also reported that the mGPS is not a significant prognosticator in a multivariate analysis (*p* = 0.06) [26]. Many results favoring the mGPS as a significant prognostic factor in patients with CRC have been derived from studies using a similar cohort [15,27,28,29,30]. As most of these studies analyzed the mGPS as a continuous value rather than a categorical value on multivariate analysis, the score showing statistical power remains unclear [15,27,28,29,30]. In a recent systematic review, the majority (60%) of patients had an mGPS of 0, with only 25% and 14.8% of patients having an mGPS of 1 and 2, respectively [11]. In contrast, some studies evaluated a cohort in which more than 80% of the patients had an mGPS of 0, which is associated with good prognosis. Factors including geography, ethnicity, and patient characteristics can explain the diversity in the distribution of the mGPS in the study cohorts. Importantly, there may be some heterogeneity in studies in which the majority of the patients have an mGPS of 0. Considering the categorical classification used for the mGPS, this can be a confounding situation that can reduce the discrimination capability of the mGPS [31]. Although the mechanism underlying the synergistic effect of the combination of the mGPS and CAR remains unclear, our results indicate that patients with an mGPS of 0 can be further classified into good prognosis and poor prognosis groups, which is consistent with previous findings [13].

Our study has some limitations. Owing to the retrospective, single-center design of the study, selection bias may be inevitable. Nevertheless, the cohort involved patients who received standardized treatments based on consistent principles at a tertiary university hospital throughout the study period. In a recent meta-analysis, the cut-off values for the CAR ranged from 0.028 to 0.65 [32]; thus, the prognostic value of the CAR in patients with CRC cannot be compared definitely between studies. Further, this study could not provide specific cut-off values for the CAR that could be appropriate for the general population owing to differences in ethnic and patient characteristics. A clearer standard for the cut-off value is needed to expand its clinical use. Another limitation is that this study included patients who underwent blood tests within one month of surgery, regardless of the patients’ inflammatory status. However, it was difficult to accurately exclude patients with inflammation due to causes other than cancer at that point due to the retrospective study design. In addition, there may be some differences in sampling time between patients, although most of the patients underwent blood test within two weeks before surgery (median 6 days, interquartile range 3–11 days). It cannot be ruled out that the different timing of blood tests will affect our findings, and future clinical trials should control this factor in a well-designed manner. The ability to discriminate survival can be improved when our combined classification is added to the AJCC stage, which is currently used as a standard for predicting patient prognosis in patients. Nevertheless, the iAUC value of CC alone was analyzed to be about 0.58, and considering the relatively low iAUC values even in CAR or mGPS, these indicators seems to not be strong for predicting patient prognosis. Therefore, future work is needed to find more useful predictive models using serum inflammatory markers in order to be used more meaningfully in the management of CRC patients.

## 5. Conclusions

Our study showed that the combination of the mGPS and CAR had a synergistic effect, and thus, had a better prognostic accuracy than the mGPS or CAR alone in patients with CRC. This combination can be used for the risk stratification of patients with CRC in clinical trials to establish its usefulness in routine clinical practice.

## Figures and Tables

**Table 1:** Patient characteristics according to the mGPS.

Variables		mGPS 0 (*n* = 609) N (%)	mGPS 1 (*n* = 122) N (%)	mGPS 2 (*n* = 38) N (%)	*p*
Sex	Female	229 (37.6)	38 (31.1)	16 (42.1)	0.316
	Male	380 (62.4)	84 (68.9)	22 (57.9)	
Age (years)	<70	428 (70.3)	61 (50)	18 (47.4)	<0.001
	≥70	181 (29.7)	61 (50)	20 (52.6)	
ASA grade	1 and 2	526 (86.4)	97 (79.5)	27 (71.1)	0.041
	3 and 4	54 (8.9)	18 (14.8)	8 (21.1)	
	No data	29 (4.8)	7 (5.7)	3 (7.9)	
BMI (kg/m^2^)	<25	414 (68)	86 (70.5)	28 (73.7)	0.784
	≥25	184 (30.2)	35 (28.7)	10 (26.3)	
	No data	11 (1.8)	1 (0.8)	0	
CEA (ng/mL)	<5	422 (69.3)	86 (70.5)	18 (47.4)	0.019
	≥5	165 (27.1)	35 (28.7)	19 (50)	
	No data	22 (3.6)	1 (0.8)	1 (2.6)	
Tumor location	Colon	393 (64.5)	97 (79.5)	35 (92.1)	<0.001
	Rectum	216 (35.5)	25 (20.5)	3 (7.9)	
Tumor size (cm)	<7	551 (90.5)	89 (73)	20 (52.6)	<0.001
	≥7	58 (9.5)	33 (27)	18 (47.4)	
Histologic grade	G1 and G2	568 (93.3)	108 (88.5)	29 (76.3)	0.003
	G3	21 (3.4)	6 (4.9)	5 (13.2)	
	Others	20 (3.3)	8 (6.6)	4 (10.5)	
LVI	Absent	453 (74.4)	92 (75.4)	26 (68.4)	0.422
	Present	133 (21.8)	29 (23.8)	11 (28.9)	
	No data	23 (3.8)	1 (0.8)	1 (2.6)	
Complications		128 (21)	38 (31.1)	12 (31.6)	0.024
Number of retrieved LNs	Mean (SD)	22.4 (14.6)	26 (15.4)	26.8 (14.1)	0.005
AJCC stage	I	157 (25.8)	20 (16.4)	2 (5.3)	0.001
	II	184 (30.2)	47 (38.5)	21 (55.3)	
	III	268 (44)	55 (45.1)	15 (39.5)	
Chemotherapy		370 (60.8)	74 (60.7)	24 (63.2)	0.956
CAR	Mean (SD)	0.1 (0.1)	0.8 (0.8)	1.7 (1.6)	<0.001

Abbreviations: ASA, American Society of Anesthesiologists; BMI, body mass index; CEA, carcinoembryonic antigen; LVI, lymphovascular invasion; LN, lymph node; CAR, C-reactive protein/albumin ratio; mGPS, modified Glasgow prognostic score.

**Table 2:** Univariate analysis of the factors associated with overall survival.

Variables		HR (95% CI)	*p*
Sex	Female	1	
	Male	1.54 (1.11–2.13)	0.008
Age (years)	<70	1	
	≥70	3.51 (2.61–4.73)	<0.001
ASA grade	1 and 2	1	
	3 and 4	1.29 (0.81–2.07)	0.279
	No data	0.91 (0.47–1.74)	0.778
BMI (kg/m^2^)	<25	1	
	≥25	0.65 (0.46–0.93)	0.018
	No data	1.14 (0.42–3.09)	0.793
CEA (ng/mL)	<5	1	
	≥5	1.90 (1.40–2.56)	<0.001
	No data	1.25 (0.51–3.08)	0.622
Tumor location	Colon	1	
	Rectum	0.91 (0.66–1.25)	0.58
Tumor size (cm)	<7	1	
	≥7	1.46 (1.0–2.12)	0.045
Complications	No	1	
	Yes	1.40 (1.02–1.94)	0.037
Histologic grade	G1 and G2	1	
	G3	1.36 (0.69–2.67)	0.364
	Others	1.58 (0.86–2.92)	0.139
LVI	Absent	1	
	Present	1.71 (1.24–2.36)	<0.001
	No data	0.85 (0.34–2.09)	0.732
Number of retrieved LNs	<12	1	
	≥12	0.65 (0.46–0.92)	0.017
AJCC stage	I	1	
	II	2.04 (1.22–3.40)	0.006
	III	3.18 (1.97–5.13)	<0.001
Chemotherapy	No	1	
	Yes	0.78 (0.58–1.05)	0.109
mGPS	0	1	
	1	1.35 (0.92–1.96)	0.118
	2	2.32 (1.38–3.90)	0.001
CAR	Low	1	
	High	1.97 (1.47–2.64)	<0.001
Combined classification	Group 1	1	
	Group 2	2.43 (1.60–3.69)	<0.001
	Group 3	1.54 (1.04–2.27)	0.028
	Group 4	2.65 (1.56–4.49)	<0.001

Abbreviations: HR, hazard ratio; CI, confidence interval; ASA, American Society of Anesthesiologists; BMI, body mass index; CEA, carcinoembryonic antigen; LVI, lymphovascular invasion; LN, lymph node; mGPS, modified Glasgow prognostic score; CAR, C-reactive protein/albumin ratio.

**Table 3:** Multivariate analysis of the factors associated with overall survival.

Variables		HR (95% CI)	*p*
Sex	Female	1	
	Male	1.41 (1.01–1.97)	0.038
Age (years)	<70	1	
	≥70	3.21 (2.35–4.38)	<0.001
BMI (kg/m^2^)	<25	1	
	≥25	0.69 (0.49–0.99)	0.045
	No data	1.07 (0.38–2.97)	0.886
CEA (ng/mL)	<5	1	
	≥5	1.43 (1.04–1.96)	0.024
	No data	2.22 (0.88–5.57)	0.087
Complications	No	1	
	Yes	1.36 (0.98–1.90)	0.063
Number of retrieved LNs	<12	1	
	≥12	0.54 (0.37–0.77)	<0.001
AJCC stage	I	1	
	II	1.93 (1.12–3.31)	0.017
	III	3.41 (2.04–5.69)	<0.001
Combined classification	Group 1	1	
	Group 2	1.47 (0.95–2.29)	0.083
	Group 3	1.14 (0.76–1.70)	0.504
	Group 4	1.90 (1.09–3.30)	0.022

Abbreviations: HR, hazard ratio; CI, confidence interval; BMI, body mass index; CEA, carcinoembryonic antigen; LN, lymph node.

**Table 4:** Comparison of C-index between Stage and Stage plus CC.

	Stage	Stage + CC
C-index (95% CI) (bootstrapped)	0.615 (0.579–0.65)	0.651 (0.612–0.689)
	*p* = 0.0056	

C-index: Harrell’s concordance index; CI: Confidence Interval.
